# The Cause of Influenza-pectoralis of Horses*Condensed and translated by A. W. Clement, V.S., from Virchow’s Archives.

**Published:** 1887-07

**Authors:** Johann W. Schutz

**Affiliations:** Professor in the Royal Veterinary School, Berlin


### ART. XXVI.—THE CAUSE OF INFLUENZA-PECTORALIS OF HORSES.*

BY JOHANN W. SCHUTZ, M. D.

*Professor in the Royal Veterinary School, Berlin.*

In 1882 Siedamgrotzky divided the inflammatory chest diseases in horses into three parts.

1st, a true catarrhal affection (Bronchitis, Broncho-pneumonia.) 2d, a true inflammatory affection (Croupous pneumonia and pleurisy. 3d, the Infectious pneumonia.

He believed the infections to be due to the influence of an outside agent, which, acting upon the lungs, produced a local inflammation, from which a severe progressive inflammation of the lung tissue and pleura arose, as well as an inflammatory hyperplasia of the bronchial glands, and an

*Condensed and translated by A. W. Clement, V.S., from Virchow's Archives.

infection of the blood and storing up of the material in the parenchymatous organs.

In an infectious pneumonia, the disease of the lung is shown through a croupous-hæmorrhagic exudation, "such as is not seen in a true croupous pneumonia," and that in the inter-lobular tissue an inflammatory œdema, and in the bronchial glands an inflammatory tumification exists.

The infectious nature of the pleuritis is shown by the presence of loosely held together coagula of citron yellow color, in the exudate, in which micrococci are constant, which are not found in the exudate of true croupous pneumonia.

He further divided the infectious pneumonia into,

1st. Inflammations of the lung, due to foreign bodies. These generally produce little inflammatory "croupous" spots in the lungs, and which contain loosely held together micro-organisms (formed ferments), which soon contaminate the softer tissues, and the diseased portion extends further and further.

2d. The inflammation of the lungs commonly found in stall-fed horses; those old horses kept for a long time in the stall. They are especially subject to bronchial catarrh, due to colds. This predisposes them to the infectious pneumonia due to the foul ferments of the over-crowded stable.

3d. The special pleuro-pneumonia which affects young horses in the spring time, which he says begins as a broncho-pneumonia, and from the action of the ferment above spoken of, takes on an infectious form.

4th. Influenza-pectoralis, which only differs from the others in that it is contagious as well as infectious, and that with the exchange of respired air, other animals may become infected.

"On account of the specific nature of the infectious material, it frequently seems hardly necessary to have a preceding catarrh, or the catarrh may follow the working of the contagion itself."

Siedamgrotzky asserts, that as to the nature of the infection of the above mentioned stall miasm, we can only say that in every case the same forms of micro-organisms appear. Most of them are micrococci, which are constant in the croupous-hæmorrhagic exudate, and also found in the inter-lobular tissue, and in the pleuritic exudate, but there are occasionally characteristic bacteria to be seen also. As it seems to be more intensive amongst horses closely stalled, perhaps the infecting material takes on this intensity, by passing through the body of an animal.

He distinguishes, also, between a simple and an infectious pneumonia as follows: By the first we have a fibrinous, and by the second a hæmorrhagic fibrinous pneumonia ; moreover, by the first, a slight fibrinous, and by the second a severe sero-fibrinous pleuritis.

Friedberger differentiates between two diseases; between influenza-pectoralis and what he terms the lobar or croupous pneumonia of horses.

The influenza-pectoralis is characterized by the following processes: The pleura furnishes an abundant and often deadly sero-fibrinous exudate. The pleurisy follows a lobular, and in most cases, multiple pneumonia, which ends in necrosis of the diseased lung tissue. Even where these pneumonic spots are quite isolated and only to be found of small size, the infectious involvment of the pleura is often very great.

The croupous pneumonia of horses is analagous to the croupous pneumonia of man. It is a specific infectious disease, and it may affect only one lung. The hepatization must become quite solid, as from the inflammatory exudation the involved tissue is rather hard to break. The cut surface has a general granular appearance. It alternates irregularly between greyish-yellow and greyish-red colored parts, with some dark-red or black-red parts. The inter-lobular tissue seems, especially in the peripheral parts of the inflamed area, plainly infilterated with serum and salts.

In this croupous pneumonia, the pleurisy at the same time present shows no tendency to progress, and especially exists without abundant exudation.

It is interesting to note that Friedberger, early in 1874, found oval bacteria, single and in chains, in the exudate of the pleural cavity. These micro-organisms, he said, grew in the lungs, produced infarcts, and through these reached the pleura, and set up a pleuritis. He spoke of this as a pleuro-pneumonia mycosis. The micro-organisms in the pleural exudate often lay upon, and in the white blood corpuscles. In the diseased portion of the lungs these micro-organisms swarmed. On the other hand, in other portions of the lung and in the pleural exudate he found "Bacterium termo and bacterium subtilles," as in fermenting masses.

Friedberger, later on, declared : By coloring with hamotoxyline, clearly defined sphero-bacteria could be found, and were seen many times in the alveoli filled with fibrinous nets. Whether these were pathogenic or not he would not say.

What Siedamgrotzky calls croupous (simple) pneumonia and pleuritis, Friedberger describes as lobar croupous pneumonia. In reference to the influenza-pectoralis, on the other hand, both authors quite agree. I must emphasize the fact that accordingto Friedberger and Fröhner, croupous pneumonia as well as influenza pectoralis is an infectious disease, and that these investigators do not either of them recognize a simple, non-infective croupous-pneumonia.

Lustig divides the lung affections of horses into catarrhal and pneumonic, and speaks of a pleuro-pneumonia form of the latter. He considers the cases which Friedberger describes as lobar-croupous pneumonia, as cases of influenza-pectoralis. He inoculated a portion of the inflamed lung, some pleuritic exudate, some fluid from the nostril, some blood and some urine from a case of influenza, on gelatine, horse blood serum, and on potatoes, and obtained a pure culture yellow in color, which, *en masse*, was of a bright yellow to citron color, according as the gelatine was thicker or thinner. At first the surface was clouded and granular, but afterwards became white. It grew very slowly, faster on gelatine than on horse blood serum. They grew in stick cultures, only on the surface or in the fissures, where the gelatine was fissured. The organisms were œrobic. They were small ovoid bacilli; after coloring with gentian violet, they looked like micrococci or diplococci. By the Ehrlich or Ribbert method they were seen to be bacilli. They were abundant in the parmchymatous exudate of the diseased portion of the lung, in the fluid pleuritic exudate, and in the blood. From the blood, Lustig obtained six pure cultures from the same case. The same for the pleuritic exudate. A gelatine culture was made fluid, and injected under the skin of a four-year old horse. A severe inflammatory œdema followed the inoculation; later, the swelling broke. It contained a yellow-colored fluid. Lustig considered these ovoid bacilli as the contagion of influenza-pectoralis.

Dickerhoff believes that the influenza of horses find its analogyy in the croupous pneumonia of man, but, as with him, the disease seldom arises of itself; it must be attributed to the same infectious cause as the pleuritis. There the infectious material is not known, so it must remain to judge the influenza-pectoralis according to the same characters, and especially according to the progress of the individual cases. The separation of croupous-pneumonia, or pneumo-pleuritis, and influenza-pectoralis, as distinctive diseases, is not permissible. The working of the contagium does not show itself according to a definite scheme. The modifications in the character of the disease are sometimes so variable, that one seeks to withhold the influenza for a group of more infectious pneumonias, with different sorts of contagion. The continued observation of the disease for many months will show this hypothesis to be unpermissible. It proves much more than a specific contagion lies at the bottom of influenza, of which the virulence from an unknown cause is sometimes stronger, sometimes weaker, and which, from this especial characteristic, sometimes single disease processes, unlike the more severe, make their appearance. The nature of the infection of influenza-pectoralis is more like the pleuro-pneumonia of cattle.

Dickerhoff further observes ,that what Friedberger decribes as lobar-croupous-pneumonia should be reckoned as influenza.

I made some investigations in 1882, but I did not finish my work, because from an anatomical standpoint alone, my conclusions would not be accepted, viz: That there was only one genuine inflammation of the lungs in horses the cause of which is generally distributed, but which only works under certain circumstances in the lung, and which is known to vary in the degree of its working. At this time I began my first bacteriological investigation, and I hoped, moreover, that proceeding according to the methods advanced by Koch, my conclusions would become known. How far I was successful, further work will show.

INVESTIGATIONS.

Body of seven or eight year old horse. The muscles yellowish-brown and soft.

Mucous membrane of intestine somewhat thickened and grey. Solitary follicles prominent. Spleen not much altered. It was soft, and on section the larger vessels filled with blood ; pulp, brownish-red. The mucous membrane of stomach covered with mucous, and in the glandular portion was greyish-brown, thick, cloudy, and somewhat granular. Liver somewhat enlarged, shining and firm ; on section it was clay-colored ; the acini were very prominent, the outer part grey-brown and cloudy, the inner dark-brown : The knife was covered with fat.

Kidneys enlarged, easily broken ; the capsule easily separated from the substance. Surface smooth, dark greyish-red and cloudy.

On section the medullary substance was dark red and cloudy. The cortical substance generally greyish-brown and cloudy.

In the plural cavity five and one-half litres of cloudy dark green fluid, in which numerous large masses of loose texture and yellow color were held. The pluera, in lower part, covered with loose yellow attachment. The surface of the pleura at this spot was covered with red granulations. In pericardium a red, but clear fluid. Many small echimoses beneath the epicardium. Under the endocardium of left ventricle a large blood clot. Heart muscle, yellowish-brown, cloudy, dry and soft. Valves normal. The lungs strongly retracted from the sternum. The anterior lobes of both lungs solid, and attachment between pleura pulmonalis and pleura costalis. On section of the solid portion the surface was dark red, smooth and moist. In this dark-colored tissue was found a cavity about the size of a hen's egg, with fluctuating contents. The contents composed of necrosed lung tissue. In this necrosed tissue many yellow loose masses lentil-sized could be seen. The dependent portions of the lungs and the larger bronchi were filled with a frothy fluid.

This autopsy showed that the horse had a "pneumonia multiple-mortificans." The cavities and yellow spots were the product of an irritant, which had affected the lung in different parts at the same time. The irritant must have been very severe to have caused this necrotic inflammation. Moreover, it must itself have progressed and become installed in the neighborhood to have set up a new inflammation which caused the death of the animal.

The cause must have exerted an influence on the blood as shown by the clouded appearance of the parenchyma.

From my experimental investigation on the Swine plague, I came to the conclusion that the cause of influenza-pectoralis must be a micro-organism, which through the respired air was carried into the lungs, and having arrived at a certain part, located itself and from there progressed further and further.

In a second post mortem similar lesions were found.

In a third, there were no yellow necrotic spots, no cavities, and no pleuritis. The right lung was dark red in color and the left gray. On section of the right lung, the dark red tissue, was beset with sharply circumscribed gray red finely granular and dryer parts. The changes in the other organs were the same as in cases one and two.

Notwithstanding, that there were no necrotic spots and no pleuritis, I shall be able to show that this was infectious pneumonia, "influenza-pectoralis."

I have discussed the well-defined alterations in the lungs of horses, which exhibit this granite appearance, in my work on the "genuine lung inflammation of horses," and I repeat that the red particles which are to be seen in the hæmorrhagic stage, and the gray or gray-red seen in the hepatized stage are indicative of influenza-pectoralis.

From cases one and two, cover glass preparations were made from the yellow spots which lay in the neighborhood of the hepatized portions, and from the exudate mass in the plural cavities. Colored with gentian violet, only oval micro-organisms were found, which were relatively small, and which were surrounded by a substance which sometimes colored, sometimes did not. The organisms lay, sometimes in pairs, sometimes single, sometimes in masses. In hardened sections of lung the same organisms were found; in the contents of of the alveoli, and in the yellow spots they were especially numerous, while on the other hand, they were less numerous in the dark red tissue, and grew less and less numerous as the healthy tissue was approached.

These observations leave no doubt in my mind, that the yellow areas were where the disease first started, and that the masses of dead tissue were due to the progressive invasion of the bacteria.

The lung tissue in case three was also examined, and the same organisms found in the contents of the alveoli, and of the bronchi and trachea.

I make this statement to show that there is no difference from an etiological stand point between influenza-pectoralis and lobor croupous-pneumonia.

A number of bodies were examined, and these micro-organisms always found.

### Pure Cultures.

On gelatine, globe-shaped masses of a white color. They do not make gelatine fluid. On the surface they take the form of little chains.

In flesh infusion they grow mostly in form of chains.

On surface of agar agar are smaller cloudy, gray masses. On the bottom of the glass they appear as gray white deposits.

Stick culture in agar agar grows the same as in gelatine.

With pieces of lung the following animals were inoculated.

| | |
|---|---|
| 2 Rabbits in the ear. | 4 White rats in back. |
| 2 Guinea pigs in belly. | 1 Hen in the wing. |

The inoculation of the rats was carried on to the fourth generation, and all died. Oval bacteria found in all the organs and in the blood. The rabbits took the disease and died. They showed the characteristic bacteria.

The guinea pig and the hen did not take the disease.

In the cavities in horses' lungs, putrefactive bacteria were found in addition to the oval bacteria.

At one post mortem, the fluid was drawn by tapping the chest, and immediately examined. The oval bacteria were found, but in addition, another organism, a chain micrococcus. The chain which these organisms formed, extended from one side of the field to the other in cork-screw fashion. I thought at first, that these cocci gained entrance by the puncture of the chest wall, but I soon came to another conclusion. By the examination of the right lung and the part which lay enclosed in the cavity, I saw that the bacteria of influenza-pectoralis were only in the dead tissue parts of the cavity, while these chain building micrococci were found in great numbers in the pus. Moreover, the thick fluid in the cavity, contained a mixture of both forms, and no other organisms could be found in the cavity.

Through the invasion of the bacteria of influenza-pectoralis, were the necrotic areas produced, and around this part later a sharp line of demarkation was produced. One part which lay near the surface of the lung showed perforation of the pleura, caused by the dissecting suppuration, and in this way entrance was gained into the plural cavity, and pluritis produced. I leave the question open as to whether this micrococcus is identical with the micrococcus pyogenes or not.

It is necessary to differentiate between the process, which brings about the necrosis of the lung tissue, and that which causes the suppuration of the necrotic parts above mentioned. The one is through the bacteria of influenza, and the other through the chain building micrococci.

Both organisms enter the lungs by the respired air, and if there is not suppuration of the lung tissue in every case, the micrococci are not distributed over the whole lung but only in certain parts, or there is not in every case, communication with a bronchus, shows through which the introduction of the organism is alone possible.

A rat, inoculated with the twelfth generation of the bacteria of influenza, died on the following day. Some of his blood was introduced into peptone beef infusion and allowed to stand three days at 358C. By this time the fluid contained a mass of characteristic bacteria. From this culture a two year old horse was inoculated, and in nine days after the inoculation, died of lung trouble.

The post mortem revealed the characteristic lesions, and the special bacteria were present.

Later, two more horses were inoculated with like result.

